# Ocular biometric responses to simulated polychromatic defocus

**DOI:** 10.1167/jov.24.12.3

**Published:** 2024-11-05

**Authors:** Sowmya Ravikumar, Elise N. Harb, Karen E. Molina, Sarah E. Singh, Joel Segre, Christine F. Wildsoet

**Affiliations:** 1 Herbert Wertheim School of Optometry and Vision Science, University of California, Berkeley, Berkeley, CA, USA; 2 Google X, Mountain View, CA, USA

**Keywords:** myopia, longitudinal chromatic aberration, ocular biometry

## Abstract

Evidence from human studies of ocular accommodation and studies of animals reared in monochromatic conditions suggest that chromatic signals can guide ocular growth. We hypothesized that ocular biometric response in humans can be manipulated by simulating the chromatic contrast differences associated with imposition of optical defocus. The red, green, and blue (RGB) channels of an RGB movie of the natural world were individually incorporated with computational defocus to create two different movie stimuli. The magnitude of defocus incorporated in the red and blue layers was chosen such that, in one case, it simulated +3 D defocus, referred to as color-signed myopic (CSM) defocus, and in another case it simulated −3 D defocus, referred to as color-signed hyperopic (CSH) defocus. Seventeen subjects viewed the reference stimulus (unaltered movie) and at least one of the two color-signed defocus stimuli for ∼1 hour. Axial length (AL) and choroidal thickness (ChT) were measured immediately before and after each session. AL and subfoveal ChT showed no significant change under any of the three conditions. A significant increase in vitreous chamber depth (VCD) was observed following viewing of the CSH stimulus compared with the reference stimulus (0.034 ± 0.03 mm and 0 ± 0.02 mm, respectively; *p* = 0.018). A significant thinning of the crystalline lens was observed following viewing of the CSH stimulus relative to the CSM stimulus (−0.033 ± 0.03 mm and 0.001 ± 0.03 mm, respectively; *p* = 0.015). Differences in the effects of CSM and CSH conditions on VCD and lens thickness suggest a directional, modulatory influence of chromatic defocus. On the other hand, ChT responses showed large variability, rendering it an unreliable biomarker for chromatic defocus-driven responses, at least for the conditions of this study.

## Introduction

The typical human eye is hyperopic in infancy, with a gradual shift toward emmetropia during the first few months of life (Cook & Glasscock, 1951; Gordon & Donzis, 1985; Mutti et al., 2005; Pennie, Wood, Olsen, White, & Charman, 2001; Wood, Hodi, & Morgan, 1995). Emmetropization has been attributed to both passive and active (i.e., visual experience–dependent) processes that together determine the rate of early eye growth (Flitcroft, 2012; Flitcroft, 2013; Hung & Ciuffreda, 2000; Mutti, Zadnik, & Adams, 1996; Wallman & Winawer, 2004; Wildsoet, 1997). Experimental studies involving a variety of different animal models have also convincingly demonstrated that early eye growth is sensitive to retinal image defocus (Schaeffel, Howland, & Glasser 1988; Schaeffel & Howland 1991; Chakraborty, Ostrin, Benavente-Perez, & Verkicharla, 2020; Smith, Hung, & Arumugam, 2014; Troilo et al., 2019). Specifically, eyes fitted with negative lenses (i.e., experiencing hyperopic defocus) show accelerated growth, whereas those fitted with positive lenses (i.e., experiencing myopic defocus) show slowed growth (Arumugam, Hung, To, Sankaridurg, & Smith, 2016; Park, Winawer, & Wallman, 2003). However, in the young, still growing human eye, the specific visual cues or combinations of cues that inform the correct direction of growth in the presence of retinal image defocus remain a subject of much debate (Flitcroft, 2012; Schaeffel & Wildsoet, 2013).

In addition to changes in axial length (AL) in response to imposed optical defocus, bidirectional changes in choroidal thickness (ChT) in response to imposed optical defocus have been reported in both animal and human studies (Ostrin et al., 2023). The latter observations have led to the emergence of ChT as a potential biomarker of retinal image defocus (Aldakhil, 2021; Delshad, Collins, Read, & Vincent, 2020; Ostrin et al., 2023; Zhu, Park, Winawer, & Wallman, 2005). For example, in humans, viewing a stimulus through a positive lens is reported to cause the choroid to thicken (Chiang, Phillips, & Backhouse, 2015; Read, Collins, & Sander, 2010), whereas viewing through a negative lens causes the choroid to thin (Chiang et al., 2015). The choroid has also been reported to thicken in response to multifocal soft contact lenses, which are among the commonly prescribed myopia control treatments (Prieto-Garrido, Villa-Collar, Hernandez-Verdejo, Alvarez-Peregrina, & Ruiz-Pomeda, 2022).

In a well-focused human eye, the largest source of retinal image blur is typically longitudinal chromatic aberration (LCA) (Campbell, Harrison, & Simonet, 1990; McLellan, Marcos, Prieto, & Burns, 2002; Vinas, Dorronsoro, Cortes, Pascual, & Marcos, 2015). Because shorter wavelengths are focused in front of longer wavelengths, contrast differences experienced by the three cone classes—short (S)-, middle (M)-, and long (L)-wavelength–sensitive cones—have been proposed as a potential cue for the sign (i.e., direction) of defocus (Foulds, Barathi, & Luu, 2013; Gawne, She, & Norton, 2022; Rucker, 2013; Rucker & Wallman, 2009; Rucker & Wallman, 2012; Schaeffel, Troilo, Wallman, & Howland, 1990; Seidemann & Schaeffel, 2002; Wildsoet, Howland, Falconer, & Dick, 1993). Thus, when an eye experiences myopic defocus, the contrast signal derived from M- and L-cone inputs will be higher than that from the S-cone input. Conversely, for an eye experiencing hyperopic defocus, the contrast signal derived from the S-cone input will be higher than that derived from M- and L-cone inputs (Rucker & Wallman, 2012).

There is some evidence that the eye can make use of color-specific changes in contrast to adjust its focus, at least in the short term, from studies of ocular accommodation (Chin, Hampson, & Mallen, 2009; Cholewiak, Love, Srinivasan, Ng, & Banks, 2017; Seidemann & Schaeffel, 2002). To study the role of LCA in stimulating ocular accommodation response, Cholewiak et al. (2017) simulated the blur predicted to arise for the three primaries in a display viewed through a defocusing lens. In brief, each primary was computationally rendered to incorporate blur, as expected due to chromatic difference in focus (termed ChromaBlur). The simulated blur stimuli were found to generate sign-appropriate accommodation responses comparable to those observed with real (optical lens–induced) changes in accommodative demand, suggesting that ocular accommodation can use LCA as a directional defocus cue.

In the study reported here, we aimed to examine the effects of simulated hyperopic and myopic defocus on key ocular biometric parameters in relation to the development of myopia and its control. Directional (i.e., sign-dependent differences in simulated defocus) were achieved by individually rendering the red and blue color channels of an original red, green, and blue (RGB) video with sign-appropriate defocus, thereby generating color-signed defocus video stimuli. Based the finding as described above, that ocular accommodation utilizes chromatic aberration as a cue (Cholewiak et al., 2017) and that imposing defocus using optical lenses in the presence of the eye's own natural LCA yields changes in ChT (Chiang et al., 2015; Read et al., 2010), we speculated that the large differences in defocus in the blue and red channels of an image *alone* could be sufficient to drive short-term changes in either ChT or AL, or both. Results consistent with this hypothesis (i.e., that the chromatic cues can drive biometric changes, at least in the short term) have been demonstrated using the chick model (Rucker & Wallman, 2012), although short-term changes in ChT and AL have not yet been unequivocally tied to longer term, myopic growth in human eyes. In designing the video-based visual stimuli to test the above hypothesis in the current study, due consideration was given to the need for the stimuli to be tolerable to viewing over an extended period, taking into consideration that beyond the current study they may have broader application as tools for myopia research. For these various reasons, we chose to leave the green channel focused while simulating defocus, either myopic or hyperopic, in the red and blue color channels. As biometric indices, both AL and ChT changes were evaluated both before and immediately after viewing unaltered (reference) and modified videos. We anticipated that the direction of biometric changes would depend on the color-sign of the viewed stimulus.

## Methods

### Implementation of color-signed blur

**Figure 1.**
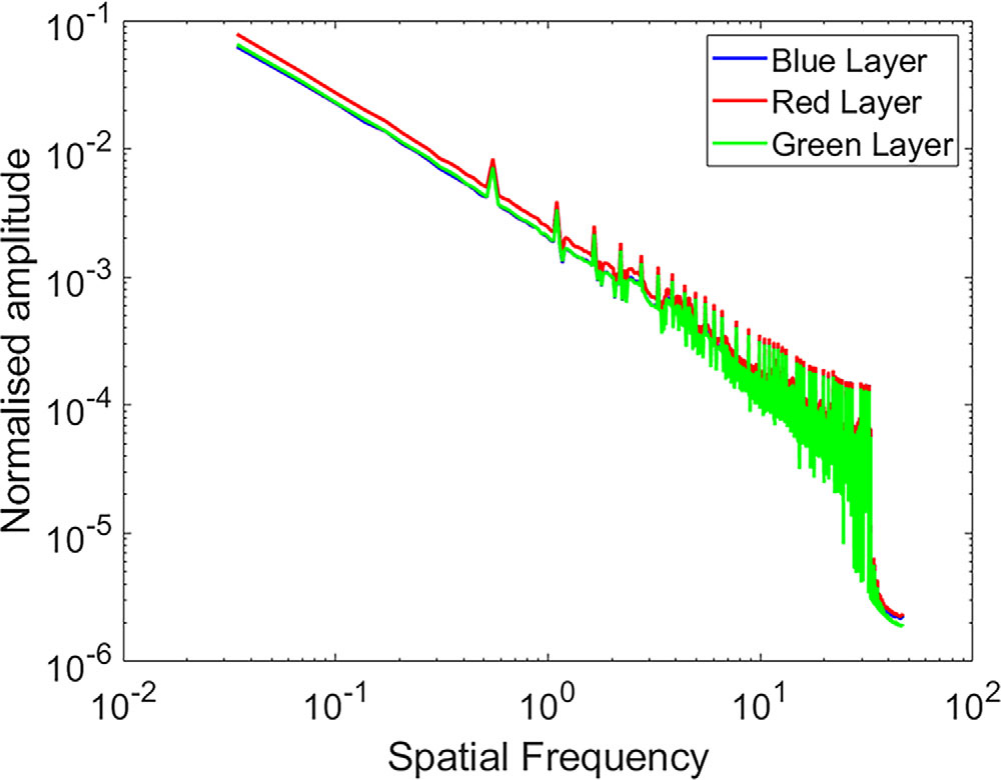
Log normalized amplitudes as a function of log spatial frequency for the red (R), green (G), and blue (B) color channels of the original stimulus video, prior to computational blurring. Mean amplitudes were derived from separate Fourier analyses of the R, G, and B layers of 71,143 frames, shown here in red, green, and blue, respectively.

The stimuli used in this study were modified versions of the Netflix documentary *One Planet* (Fothergill & Keith, 2019). This video was chosen based on the broad spatial frequency spectra of most frames (Figure 1) and, due to its dynamic content, its ability to capture the attention of participants for an extended (hour-long) period. The goal of the rendering pipeline was to generate retinal images corresponding to the R and B channels, as would be experienced by an eye subjected to either +3 or −3 diopters (D) of optical defocus. We refer to the latter as the *desired retinal defocus* (DRD). The magnitude of 3 D was chosen based on previous studies reporting significant ChT changes under the same defocus conditions (Hoseini-Yazdi, Vincent, Collins, & Read, 2019; Read et al., 2010; Wang et al., 2016). In the regime of simulated polychromatic defocus, the sign of defocus is implied by the ratio of defocus experienced by the R and B retinal images. Because the ocular LCA of the observer's eye also contributes differential defocus of the R and B channel images, the respective magnitude of defocus due to LCA must be subtracted from the DRD to calculate the amount of defocus that must be computationally imposed in each channel (R and B) of the experimental stimuli. We refer to the latter as the *imposed computational defocus* (ICD) (Equation 1). The value for ocular LCA as described by the Indiana eye chromatic aberration function (Thibos, Ye, Zhang, & Bradley, 1992) was used in these calculations. In both experimental stimuli, the green channel was kept in focus (i.e., DRD for 550 nm was set to 0).

**Figure 2.**
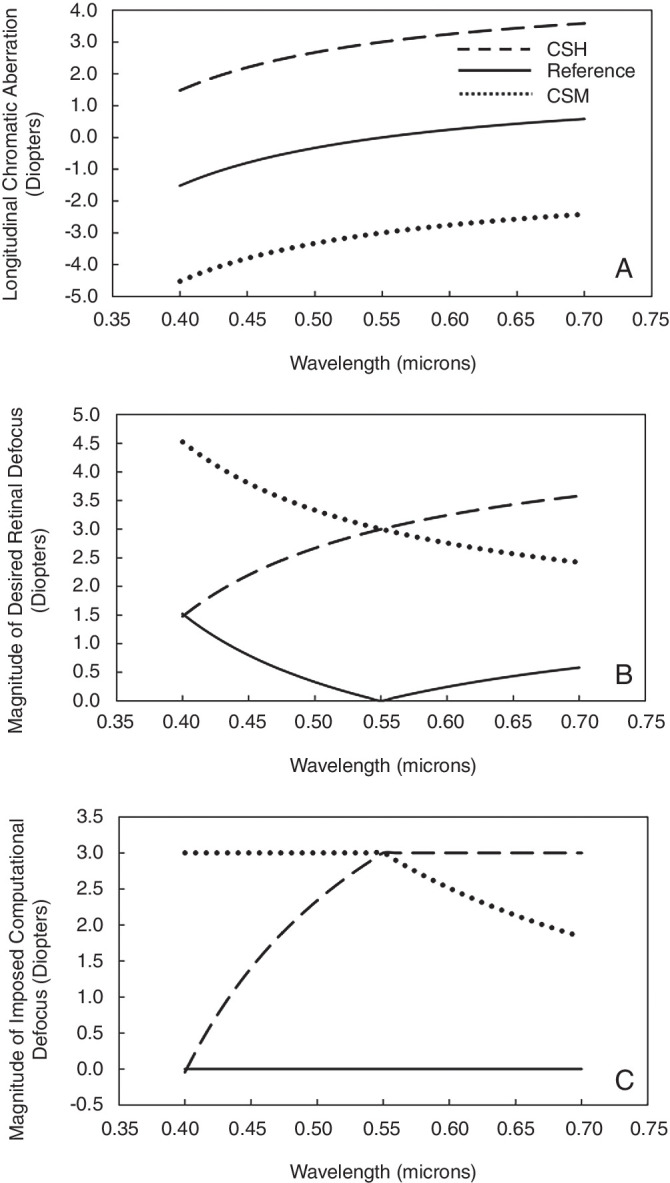
Panels show the components of Equation 1. (**A**) LCA as a function of wavelength for an emmetropic eye (solid black line), 3-D myope (dotted line), and 3-D hyperope (dashed line). (**B**) Magnitude of desired retinal defocus as a function of wavelength for the reference condition (solid black line), CSM condition (dotted line), and CSH condition (dashed line). (**C**) Magnitude of imposed computational defocus as a function of wavelength used to generate the stimuli for the CSM condition (dotted line) and the CSH condition (dashed line). No computational defocus was imposed in the reference case (solid black line)*.*

Based on Equation 1, a customized chromatic function was created for the color-signed myopic (CSM) and color-signed hyperopic (CSH) conditions that dictated the amount of *imposed computational defocus* (ICD, in diopters) to be applied to each wavelength (Figure 2).

(1) $$\begin{array}{c} ICD_{\left(\lambda \right)}=DRD_{\left(\lambda \right)\;}-\;\left|LCA_{\left(\lambda \right)}\right|\; \end{array}$$

where λ is wavelength.

**Figure 3.**
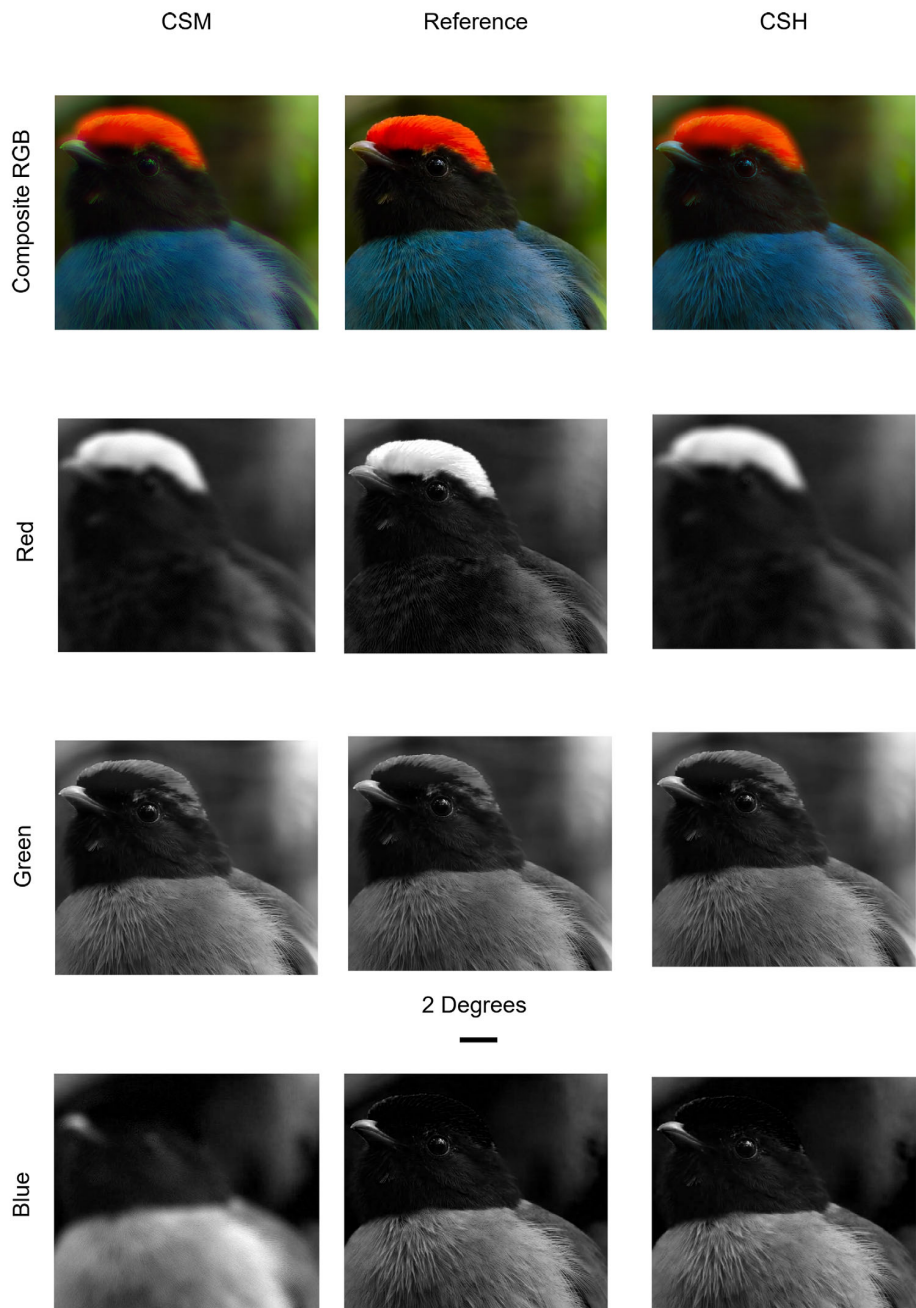
Components of a sample frame from the experimental videos. The topmost row of images shows the true color composite RGB image of the same sample frame from all three experimental conditions (reference, left panel; CSM, center panel; CSH, right panel). Isolated red, green, and blue channel grayscale images from the corresponding experimental condition are shown in the corresponding panels of the second, third, and bottom rows, respectively. Note that the isolated green channel grayscale images are invariant across the three conditions. The black scale bar below the green reference image represents an angular size of 2°.

Applying the ICD for each of the two color-signed conditions (i.e., CSM and CSH), polychromatic point-spread functions (PSFs) were calculated for R, G ,and B channels using a computational approach, Ravikumar et al. (2008) as described in detail in Appendix A. In brief, for each video frame, the composite RGB image was decomposed into its R, G, and B channels, the three PSFs were convolved with the respective channels of the image, and the outputs were recombined into a composite RGB image incorporating color-signed defocus. The composite RGB images were recompiled to generate the CSM and CSH stimulus videos. A selected portion of one frame from each stimulus video is shown in Figure 3, both as a composite and as individual R, G and B channel images. Note that, in this process, the PSF for the G channel in both CSM and CSH conditions is a diffraction-limited PSF, with no imposed defocus.

### Experimental setup

In total, three different stimulus conditions were tested in this study—a reference (unmodified) stimulus and two simulated defocus conditions, each on a different day, with each session lasting ∼1 hour. All visits occurred between 9 a.m. and 1 p.m. and at least 2 hours after waking. Subsequent visits were scheduled within an hour of the first visit for consistency, thus minimizing the impact of diurnal variations in various ocular parameters. Participants were instructed not to consume caffeine on the day of testing and to otherwise maintain their usual habits in relation to waking time and exercise. Subjects were asked to wear their soft contact lenses for at least 2 hours prior to each visit, and prior to the start of each experimental session a pre-visit questionnaire was administered to verify participant compliance with the experimental protocol (see Supplementary Materials).

Participants were pseudorandomly assigned to view one of the three different stimuli on a given day of the study: reference, CSH, or CSM. All participants completed the reference condition and at least one of the two simulated blur conditions, with eight of the 17 participants completing all three blur conditions (see Table 1)^3^. Participants binocularly viewed the stimulus videos on a widefield projection screen at a distance to 5.1 meters to minimize accommodation and corresponding to a field of view of 17° vertically and 28.5° horizontally. An 8-bit digital light projector was used to display the RGB videos, at a 1920 × 1080-pixel resolution and at a rate of 24 frames per second. Videos were accompanied by audio narrative in English, without subtitles. Immediately prior to the start of experimental viewing sessions, participants viewed an unaltered nature video (a different episode of *One Planet*) for 20 minutes as a “washout condition.” Both washout and experimental sessions made use of the same projection setup.

**Table 1.** Key features of study participants along with details of completed conditions and eye selected for biometric measurements. All participants completed the reference condition and at least one of the two simulated blur conditions; eight participants completed all three conditions. CL Rx, contact lens prescription; OD, right eye; OS, left eye.

					Conditions completed		
Number	Age (y)	Test eye	CL Rx (D)	AL (mm)	Reference	CSM	CSH
1	19	OD	−3.75	24.41	Y	Y	Y
2	23	OS	−1.75	24.64	Y	Y	Y
3	19	OS	−1.25	25.21	Y	Y	Y
4	20	OD	−4.75	25.90	Y	Y	Y
5	24	OD	−4.50	26.00	Y	Y	Y
6	25	OS	−3.75	26.05	Y	Y	Y
7	20	OS	−5.50	26.24	Y	Y	Y
8	24	OS	−2.00	26.44	Y	Y	Y
9	24	OS	−2.25	23.85	Y	Y	—
10	20	OS	−2.50	24.34	Y	Y	—
11	24	OD	−2.50	25.01	Y	Y	—
12	18	OS	−2.50	25.16	Y	Y	—
13	21	OD	−4.25	25.37	Y	Y	—
14	22	OD	−4.00	25.50	Y	Y	—
15	25	OD	−5.00	25.63	Y	Y	—
16	26	OD	−1.75	23.45	Y	—	Y
17	24	OD	−2.75	24.05	Y	—	Y

### Ocular biometric measurements

At the end of the washout period and before viewing the experimental stimuli, ocular biometric data were collected from one eye, with equivalent additional data collected from the same eye at the end of the experimental session. The test eye was chosen randomly for a given subject during their first visit, and the same eye was used in all subsequent experimental session.

Biometric measurements were made with the participants’ habitual contact lens prescription in place. Mean AL, lens thickness (LT), and vitreous chamber depth (VCD) data were generated from five good-quality measurements captured using a Lenstar LS900 optical biometer (Haag-Streit, Köniz, Switzerland). The decision to leave the contact lenses in place, although not conventional, avoided the possibility that the physical act of lens removal in and of itself could affect one or more of the measured parameters. No correction factors were applied, as all measurements of individual subjects were made with the same contact lenses in place within a given experimental session. In support of these decisions, there is also published evidence that the presence of contact lenses does not significantly alter the repeatability and reliability of biometry (Lewis, Knellinger, Mahmoud, & Mauger, 2008).

ChT data were derived from optical coherence tomography (OCT) images captured using a Triton Swept-Source Multimodal OCT (Topcon Healthcare, Tokyo, Japan). Three horizontal 9-mm, five-line raster scans centered on the fovea were used in these analyses, allowing for good visibility of the choroidal boundaries. For each of the three captured OCT scans, the line scan with the deepest foveal pit, as determined by an experienced clinician (KM), was used for analysis. TABS software developed by Topcon was used to generate segmentation lines between the inner retinal pigment epithelium border and the outer choroid–sclera interface. The deepest part of the foveal pit, manually located and marked on each image, was used to define the location of the fovea as the reference landmark for choroid thickness analysis. Custom, lab-developed software (Python) was then used by experienced clinical graders to review results from automatic segmentation and as necessary, make any corrections manually. This image analysis protocol was used to determine both central (subfoveal) ChT and additional ChT values at 100-µm intervals, both nasally and temporally from the foveal reference position. Reported subfoveal ChT values represent the average ChT across the central 1 mm (i.e., 0.5 mm nasal and 0.5 mm temporal to the foveal pit reference).

The above procedures were performed on each of the three collected horizontal scans by two of three experienced graders (EH, SS, SR), who were masked to the origin of the scan being analyzed (i.e., specific test condition). The average of these paired datasets was used in follow-up analyses, unless the data from individual graders differed by more than 15 µm, in which case an adjudication process involving the two graders was performed to reach consensus. If consensus could not be achieved (in 9 of 252 images analyzed), the third grader was brought in to adjudicate; therefore, all final measures represent the average of results from either two or three graders.

### Data analysis

Biometric data, captured immediately prior to and at the end of each stimulus viewing session, were compared for each of the completed experimental conditions, with graphical analyses performed to visualize the distribution of changes for the participant cohort. Statistical analyses made use of paired *t*-tests to examine differences within and across sessions for individual subjects, and two-way, repeated-measures analyses of variance with Bonferroni correction were used to compare induced changes corresponding to the three stimulus conditions.

## Results

A total of 17 young healthy adult females, between the ages of 18 and 26 years, participated in this study. Their profiles are summarized in Table 1 and represent the typical demographics of the recruited University of California Berkeley student population. All participants were soft contact lens wearers, with prescriptions ranging from −1.25 to −5.50 D and habitual corrected visual acuity better than 20/25. Subjects with anisometropia of 1.00 D or more were excluded. All participants were screened to ensure unremarkable ocular and medical health via a screening questionnaire (see Supplementary Materials).

**Figure 4.**
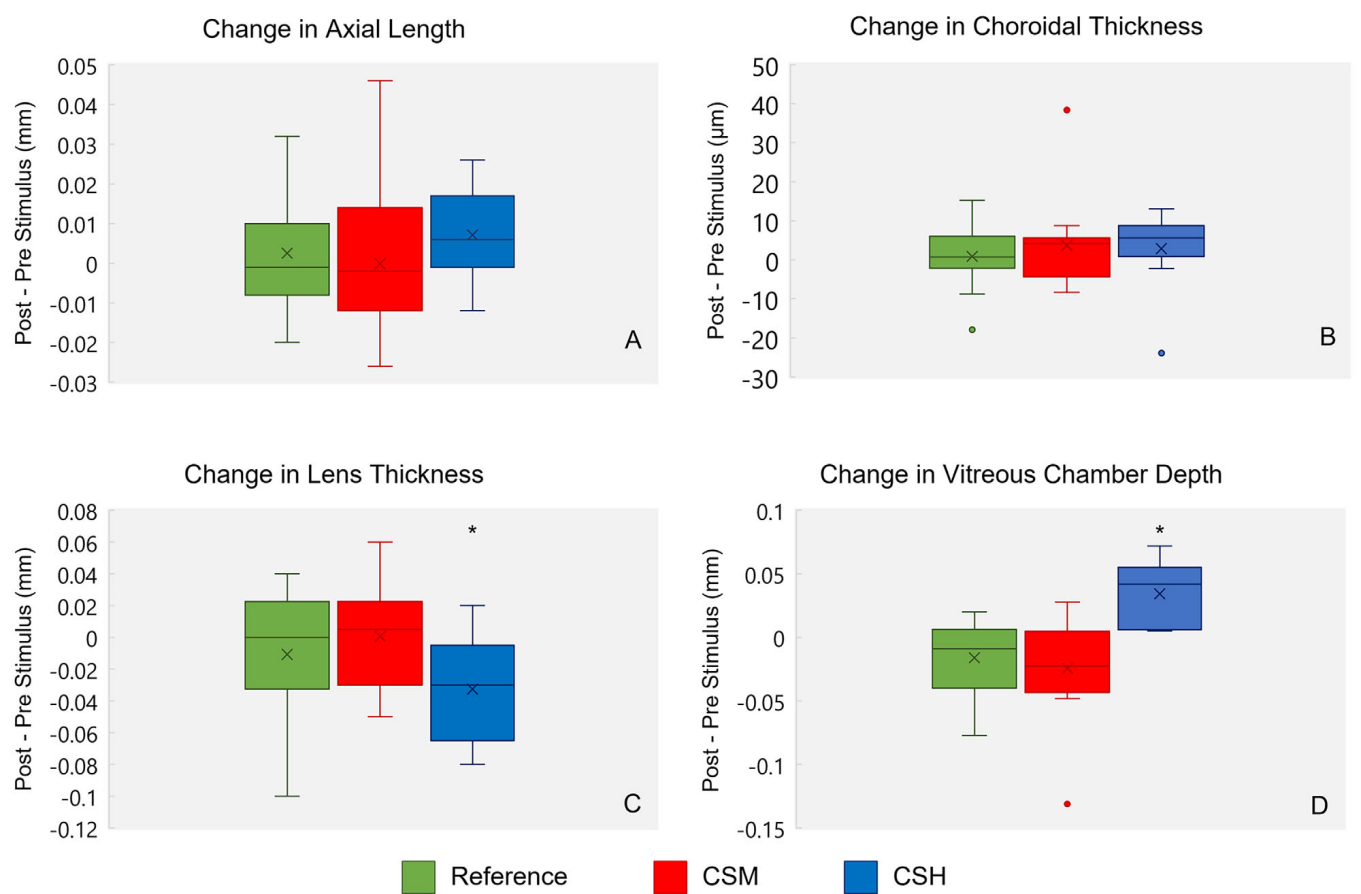
Box plots showing changes across each of the three viewing conditions (reference, green; CSM defocus, red; CSH defocus, blue), in axial length (**A**), subfoveal ChT (**B**), LT (**C**), and VCD (**D**). The horizontal line in each box represents the median change, and the X represents the mean change. Outliers are shown as dots of corresponding colors. Relative to that of the reference condition, the change in LT was statistically significant for the CSH condition (C, *p* = 0.015), as was the change in vitreous chamber depth for the CSH condition (D, *p* = 0.018).

For each of the three viewing conditions, the distribution of changes across the session, including post- and pre-stimulus changes in AL, VCD, LT, and ChT (central 1 mm), are shown in Figure 4 and include available data from all participant–condition combinations, as summarized in Table 1. For the reference condition, there was no significant change in any of the key measured ocular parameters (mean change ± *SD*: AL, 0.003 ± 0.01 mm; VCD, −0.016 ± 0.03 mm; LT, −0.011 ± 0.04 mm; ChT, 0.899 ± 7.65 µm). For each of the two experimental conditions, there was also no significant change in AL (CSH, −0.007 ± 0.01 mm; CSM, −0.0001 ± 0.02 mm) and no significant differences across all three viewing conditions in the AL changes. On the other hand, changes in VCD for both the CSH and CSM tended to be larger than those recorded with the reference stimulus and opposite in direction from each other, consistent with the sign of imposed simulated defocus. Thus, following viewing of the CSH and CSM stimuli, relative increases and decreases in VCD were recorded, respectively, albeit not significant in the latter case (CSH, 0.034 ± 0.03 mm vs. reference, 0 ± 0.02 mm, *p* = 0.018; CSM, −0.024 ± 0.04 mm vs. reference, −0.018 ± 0.03 mm, *p* = 0.35). Significant thinning of the crystalline lens was also observed with the CSH stimulus condition relative to the CSM stimulus condition (LT, −0.033 ± 0.03 mm vs. 0.001 ± 0.03 mm, respectively; *p* = 0.015), although neither of these changes was significantly different from that recorded with the reference stimulus (−0.011 ± 0.04 mm).

In relation to ChT changes, both CSM and CSH stimulus conditions led to small increases in subfoveal ChT (CSM, 3.67 ± 10.93 µm; CSH, 1.21 ± 10.95 µm), although neither of the changes was statistically significant. There was also no significant difference in the changes in ChT across the three stimuli conditions. Furthermore, although changes in ChT appeared to be less variable for CSM compared with CSH stimulus conditions (ranges, −0.6 to 6.4 µm for CSM vs. −24 to 13.5 µm for CSH), differences in sample size for the two conditions offer a plausible explanation.

**Figure 5.**
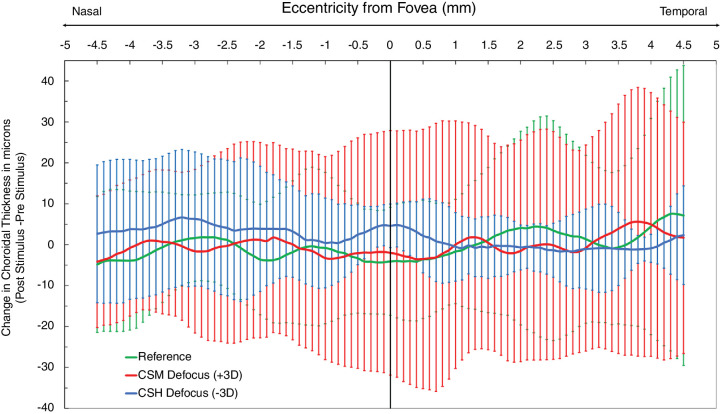
Mean changes in ChT (post- and pre-stimulus viewing), with the solid lines indicating reference (green), CSM (red), and CSH (blue), as a function of eccentricity from the foveal pit, indicated by 0 on the *x*-axis, with nasal and temporal retinal regions on either side assigned negative and positive values, respectively. Error bars represent ±*SD*.

ChT changes were further analyzed in terms of local changes within the 9-mm horizontal OCT scans. As is evident from the large error bars (2 *SD* of the mean) (Figure 5), especially at temporal locations corresponding to the results for the reference and CSM conditions the variability in observed changes is large. Nonetheless, based on mean changes, there was a trend for the CSH condition toward relative thickening, both subfoveally and at nasal locations, compared with the changes recorded with both reference and CSM conditions.

**Figure 6.**
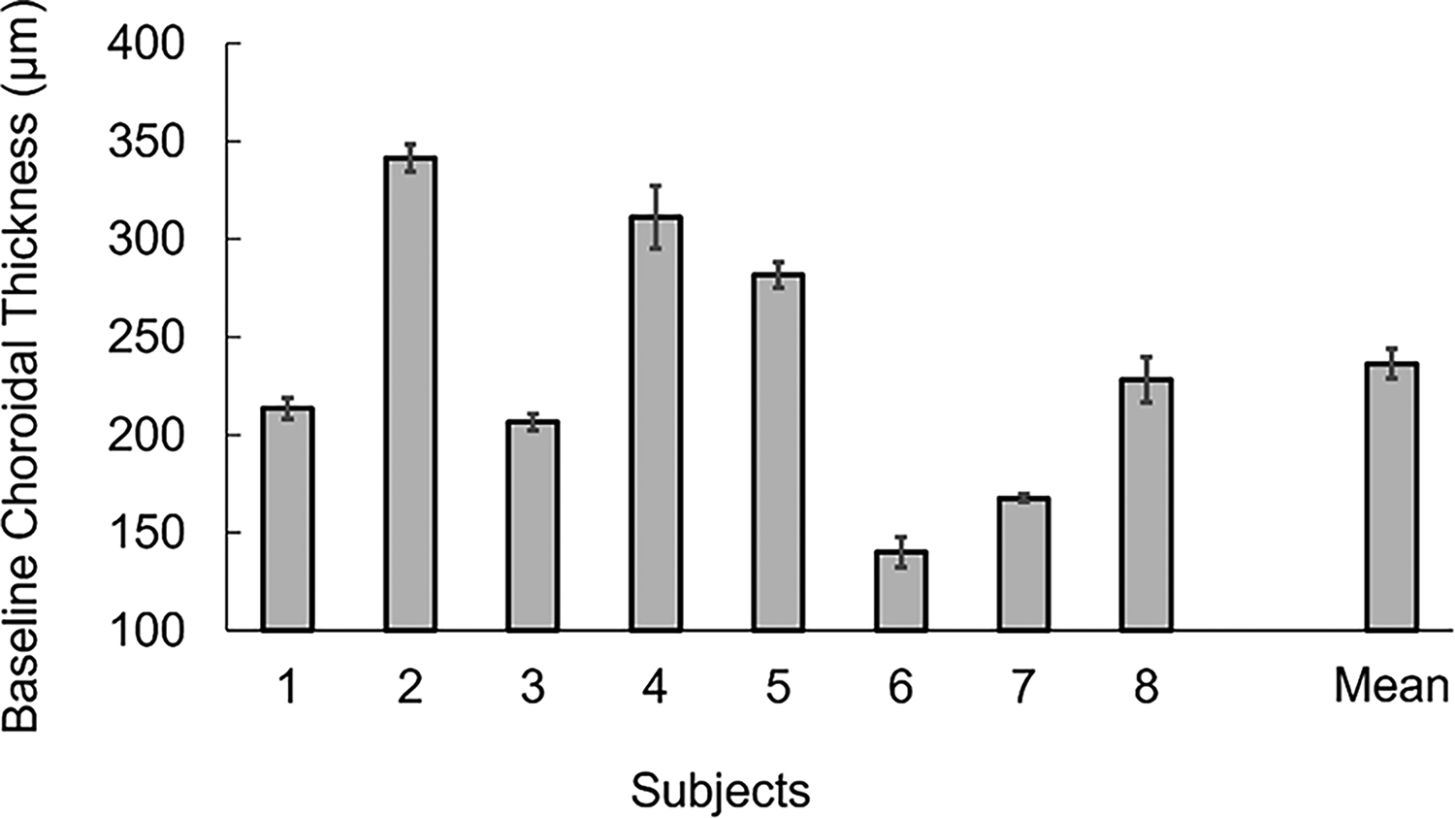
Baseline ChT, averaged across the central 1 mm and further averaged across the three study visits for the eight subjects completing all three study visits. The mean ChT for the group is shown on the right. Error bars represent 2 *SD* of the mean in all cases.

To obtain insight into the origin of the variability in ChT changes, baseline ChT values corresponding to the central 1 mm (i.e., as measured at the end of washout periods) were examined (Figure 6). For the eight participants who completed all three study visits, the range in baseline ChT, averaged across all study visits, is quite large (140–341 µm), although the mean was consistent with reported values for normal human ChT (mean, 236.26 µm; *SD* = 69.88 µm) (Ostrin et al., 2023). However, for individual subjects, the variability in baseline ChTs recorded across their three study visits is quite small (*SD* range, 1.9–16 µm).

**Figure 7.**
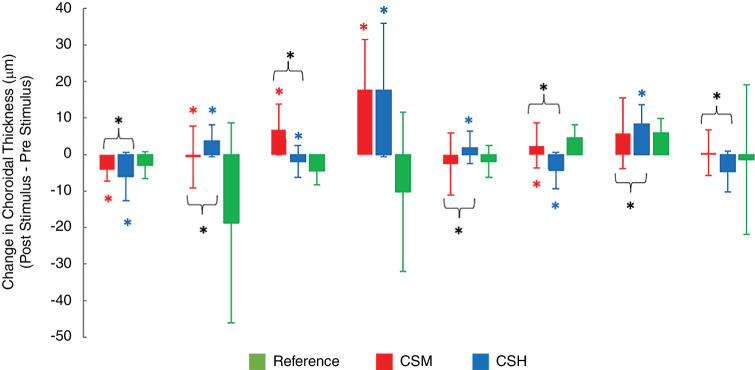
Mean change in ChT averaged across the 9-mm scan for the three different viewing conditions and each of the eight participants who completed all three test conditions. Error bars represent 2 *SD* of the mean. Red, blue, and black asterisks indicate significant differences between CSH and reference, between CSM and reference, and between CSH and CSM, respectively (*p* < 0.05).

For the eight participants who completed all three conditions, differences in their responses across these conditions (post- and pre-stimulus) were also analyzed, using ChT averages derived from the same widefield (9 mm) scans. The changes in the latter values are plotted for each participant and each of the three conditions in Figure 7. Although five out of eight participants recorded significant differences in their choroidal responses to each of the two test stimuli compared to the reference condition, there was no consistent trend across stimuli. Of the eight participants, four recorded relatively thinner choroids after viewing the CSH stimulus compared with the CSM stimulus, whereas three of the eight participants recorded relatively thicker choroidal after viewing the CSH stimulus compared with the CSM stimulus. Thus overall, although ChT appeared to be significantly altered by the color-signed stimuli, there was no consistency in the direction of changes across the subjects.

## Discussion

The purpose of this study was to determine whether simulating the effect of defocus sign-specific changes in color contrast would induce detectable differences in key biometric parameters that have been linked to ocular growth regulation. To this end, RGB videos that incorporated contrast changes in red and blue channels to simulate the effects of LCA under hyperopic and myopic defocus conditions (−3 D and +3 D, respectively) were created, and the green color channel was left unaltered (i.e., in focus). Based on the results of a previous, related study involving human ocular accommodation (Cholewiak et al., 2017), we anticipated that, relative to the reference condition, the choroid would thin and axial length increase after viewing the CSH stimulus video, which incorporated more blur in the red compared to blue channels, and that the choroid would thicken and AL would decrease after viewing the CSM stimulus video, which incorporated more blur in the blue compared to red channels. Also, because the vitreous chamber depth accounts for most of the AL of the human eye, we anticipated that changes in vitreous chamber depth would closely follow those in axial length.

In support of the above hypothesis, VCD was found to be significantly shorter after viewing the CSM stimulus video than after viewing the reference stimulus. VCD was also longer after viewing the CSH stimulus, although this change was not statistically significant. On the other hand, LT was significantly reduced after viewing the CSH stimulus but not significantly altered after viewing the CSM stimulus. In the case of the VCD result, the finding is generally consistent with the hypothesis that myopic defocus (simulated by greater blur in the blue compared to red channel) is protective against excessive eye elongation. Nonetheless, the changes in AL and ChT recorded under the three stimulus conditions (reference, CSM, and CSH) were not significantly different from each other.

In most animal and human studies investigating the impact of optical defocus on eye growth, ChT has been widely used as a potential biomarker, although its ability to predict future myopia risk remains the subject of debate (Ostrin et al., 2023). To further examine this issue in the current study, changes in ChT, averaged across 9-mm scans, as well as in the central 1-mm subfoveal ChT, in response to computationally altered stimuli were examined. Surprisingly, we did not find significant differences in the ChT responses to the three stimulus conditions. In comparison to the reference condition, both CSM and CSH conditions induced slight choroidal thickening on average, possibly due to the overall greater blur present in the color-signed stimuli compared with the well-focused reference stimulus. However, no sign-specific changes in thickness were observed in the central subfoveal choroid. Likewise, targeted analysis of ChT changed across the central 9 mm in the eight subjects tested with all three conditions, revealing directional inconsistency, even though significant sign-dependent responses were recorded for some individual participants. Below, we considered a few possibilities for the lack of significant changes in ChT and overall AL.

One of the most significant challenges in studying ChT is its inherent variability (Ostrin et al., 2023). In fact, a key reason underlying the lack of ChT change in the current study could be the large intersubject variability, as evident in the baseline ChT data for our subjects (Figure 6). Nonetheless, for any given subject, baseline ChT measurements taken at the end of the wash-out periods were quite repeatable across visits. For example, the mean intrasubject variance in ChT for the eight subjects who completed all three test conditions was 7.55 µm, which is comparable in magnitude to the repeatability reported in two other studies (Hoseini-Yazdi et al., 2019, Yazdani, Ehsaei, Hoseini-Yazdi, Shoeibi, Alonso-Caneiro, & Collins, 2021). However, although the latter value is significantly less than the intersubject variance in mean baseline ChT (∼70 µm), it is nonetheless larger than the effect sizes reported in previous studies (Chiang et al., 2015; Read et al., 2010). As comparable individual baseline ChT data are not always available for previous related studies involving imposed defocus, we cannot rule out the possibility that their subject pool may have showed less intersubject variability in baseline ChT. It must also be acknowledged that our subject pool is relatively small, and it is possible that with a larger subject pool statistically significant differences in ChT changes across our three stimulus conditions may have been observed. Nonetheless, although previous studies have used as few as 12 participants (Chiang et al., 2015) and as many as 51 participants (Wang et al., 2016), the standard deviations reported in the former study for baseline subfoveal ChT and myopic and emmetropic subgroups (i.e., 42 µm and 62 µm, respectively) are similar to values reported for the current study.

That the individual study conditions, such as duration and magnitude of induced defocus, as well as measurement schedules, vary widely among related studies also tends to rule out meaningful, direct comparison of findings in most cases. However, in relation to differences in the timing of measurements across studies, based on the results of two previous studies, we cannot rule out the possibility that choroidal responses regressed over the course of the 1-hour stimulus viewing sessions used in the current study, thereby masking earlier changes. Specifically, in two independent studies, peak choroidal responses to blur were observed over shorter time frames, close to 30 minutes into a 60-minute viewing window in one study (Chiang et al., 2018) and closer to 45 minutes in the other study (Hoseini-Yazdi et al., 2019), with slight regression thereafter.

It is possible that, if AL were measured in real time with a higher resolution technique, we could more closely examine the relative contribution of changes in ChT to AL changes. However, given the many sources of variability and noise in ChT data, with eccentricity, time-dependent variations, and the subjectivity of thickness analyses being just three examples, it is likely that this will only be achieved in the presence of much larger and more consistent changes in ChT. Nonetheless, that VCD showed more robust changes than AL likely reflects the additional sources of variability apart from ChT, such as anterior chamber depth and LT.

Other possible explanations for the differences in defocus-induced choroidal responses as reported here and in previous studies relate to specific differences in the imposed defocus conditions and the refractive error profiles of the subjects. Although computational defocus was used in the current study, previous studies have used “real” (optical) defocus (Chiang, Chen, & Phillips, 2018; Hoseini-Yazdi et al., 2019; Sander, Collins, & Read, 2018), with the one exception being a recent study by Swiatczak and Schaeffel (2021). In that study, computational blur applied to grayscale images uniformly blurred all the wavelengths that comprised the stimulus, consistent with the expected effect of optical defocus. This approach contrasts with that used in the current study, in which the green channel was intentionally left in focus, adopted with the goal of isolating the differential effects of blur in the red and blue channels, with the secondary benefit of improved visibility of the stimuli. It is possible that the well-focused middle wavelengths, close to the peak of the photopic sensitivity function of the human retina, served as a focusing anchor and that blurring the green channel would elicit larger, more consistent choroidal responses. Although ChT was not monitored in the closely related study of Swiatczak and Schaeffel (2021), significant changes in AL were reported, consistent with the imposed, simulated defocus. The latter finding lends weight to the possibility, as raised previously, that the inclusion of a focused green channel in the color-signed defocus stimuli in the current study dampened the effects of image blur in the red and/or blue channels. Given that all of the subjects in the current study were myopic, the observation in a follow-up study by Swiatczak and Schaeffel (2022) that young adult emmetropes but not myopes responded with appropriates AL changes to the simulated defocus conditions offers yet another explanation for the lack of consistent trends in the data reported here.

## Conclusions

The lack of significant changes in ChT and AL in response to our two (CSM and CSH) stimuli does not support a role for chromatic aberration as a bidirectional defocus cue guiding biometric changes. However, stimulus-dependent differences in individual VCD and LT responses offer some support to the hypothesis that color-signed, computationally induced defocus can influence biometric parameters in a directional manner. Although similar observations have been previously reported in humans with accommodation (Cholewiak et al., 2017), and in ocular growth responses in chicks (Rucker & Wallman, 2012), our study is the first, to our knowledge, to comprehensively examine ocular biometric responses to imposed chromatic blur in humans.

The computational technique used in this study has potential applications in future studies investigating the impact of chromatic aberration on ocular growth and specifically as a tool for investigating the stimuli driving myopic growth. In addition to other stimulus parameters, such as spatial frequency and duration, the influences of age and refractive error also warrant investigation in this context, given the more recent findings of Swiatczak and Schaeffel (2022).

## Supplementary Material

Supplement 1

## Appendix A Appendix A

Given the ICD function for CSM and CSH, wavefront aberration maps at each monochromatic wavelength λ were computed as *W*(*u*, *v*), where *W* is the optical path difference over pupil coordinates *u* and *v*. A pupil diameter of 4 mm was chosen for the computations.

(A.1) $$\begin{array}{c} P\left(u,v\right)=A\left(u,v\right)e^{i2\pi W\left(u,v\right)/\lambda }\; \end{array}$$

The *PSF*(*x*, *y*, λ) at image coordinates *x* and *y* is then calculated as the inverse Fourier transform of the autocorrelation of pupil function *P*(*u*, *v*)*.*

(A.2) $$\begin{array}{c} PSF\left(x,y,\lambda \right)=IFT\left\{P\left(u,v\right)⊗P\left(u,v\right)\right\}\; \end{array}$$

An array of *PSF* values was calculated for multiple wavelengths of each of the three primaries. The polychromatic *PSF* for each color primary *p* is given as

(A.3) $$\begin{array}{ccc} PSF\left(x,y,p\right)\;\; & = & \int_{\lambda =min}^{max}S\left(\lambda \right).V\left(\lambda \right)\\ & & \;\cdot PSF\left(x,y,\lambda \right)d\lambda \; \end{array}$$

where *S*(λ) is the radiance spectrum of the source, and *V*(λ) is the visual sensitivity function of the human eye.

Because the image would be viewed by an observer directly, radiance values *S*(λ) for the primaries were taken to be unit step functions. The min–max limits of *S*(λ) for the blue primary were set to 400 to 500 nm; for the green primary, they were set to 500 to 60 nm; and for the red primary, they were set to 550 to 700 nm. The limits were chosen based on radiance characteristics of DLP portable projectors (Seime & Hardeberg, 2002). For the current experiment, we employed the YABER Y30 (LED source, LCD display; Yaber Entertainment Projector, Shenzhen, China) paired with a 120-inch front projection screen (Elite Screens, Garden Grove, CA). The color look up table (CLUT) for each primary was individually gamma corrected using an IL1700 research radiometer (International Light Technologies, Peabody, MA). The maximum measured brightness of the display was 80.9 lumens/ft^2^. Note that no weighting for the human photopic visual sensitivity in the *V*(λ) function was adopted (Stockman, MacLeod, & Johnson, 1993), as the images would be directly viewed by the subject.
