# Supplementary material for: Ocular biometric responses to simulated polychromatic defocus
Source: J Vis. 2024 Nov 5;24(12):3. doi: 10.1167/jov.24.12.3 (PMC11540029; doi:10.1167/jov.24.12.3)
Supplement: Supplement 1 [file jovi-24-12-3_s001.docx]

**Participant Screening Questionnaire**

1. What is your first and last name?

- *Open ended answer*

2. How old are you?

- *Open ended answer*

3. What is your biological sex?

- *Male*
- *Female*

4. Do you have a history of systemic disease?

- *No*
- *Yes ^EXCLUDED^*

5. Do you take systemic medications? What medications?

- *Open ended answer*

6. Do you smoke?

- *No*
- *Yes ^EXCLUDED^*

7. Do you have any history of eye disease?

- *No*
- *Yes (Answer 7a)*

7a. Have you been diagnosed with any of the following conditions? (Select all that apply)

- *Dry Eye*
- *Any type of retinal pathology*
- *Glaucoma*
- *Any type of optic nerve pathology*
- *Ocular allergies*
- *Other eye disease (notate below)*
  - *Open ended answer*

8. Do you have a history of binocular vision disorders (e.g. amblyopia, eye turn/strabismus, accommodative disorders, convergence disorders)?

- *No*
- *Yes ^EXCLUDED^*

9. Do you have a history of eye surgeries?

- *No*
- *Yes ^EXCLUDED^*

10. Do you take any eye drops or medications for your eyes?

- *No*
- *Yes (Answer 10a)*

10a. What kind(s) of eye drops or medications you take for your eyes? (Select all that apply)

- *Artificial Tears*
- *Allergy / Antihistamine Drops*
- *Glaucoma Medications*
- *Steroid Medications*
- *Antibiotic Drops / Ointment*
- *Topical Immunomodulator (e.g. Restasis / Xiidra)*

11. Do you have any history of eye injuries / trauma?

- *No*
- *Yes ^EXCLUDED^*

12. Do you have any history of eye infections (not contact lens related)?

- *No*
- *Yes ^EXCLUDED^*

13. What type of correction do you use?

- *Uncorrected ^EXCLUDED^*
- *Glasses only ^EXCLUDED^*
- *Glasses & Contact Lenses*

14. Do you wear your glasses and/or contact lenses every day?

- *No ^EXCLUDED^*
- *Yes*

15. Do you wear your glasses and/or contact lenses for most of the day?

- *No ^EXCLUDED^*
- *Yes*

16. What is the prescription for your right eye?

- *Open ended answer*

17. What is the prescription for your left eye?

- *Open ended answer*

18. Are you a soft contact lens wearer?

- *No ^EXCLUDED^*
- *Yes*

19. Do you have a history of eye infections or complications related to contact lens wear?

- *No*
- *Yes ^EXCLUDED^*

20. Do you wear astigmatism / toric contact lenses?

- *No*
- *Yes ^EXCLUDED^*

21. What brand of contact lenses do you wear?

- *Open ended answer*

22. What is your contact lens prescription for your right eye?

- *Open ended answer*

23. What is your contact lens prescription for your left eye?

- *Open ended answer*

24. Is your best corrected visual acuity with glasses 20/20 or best corrected with contact lenses better than or equal to 20/25?

- *No ^EXCLUDED^*
- *Yes*

25. What time on average do you wake up in the morning (24:00)?

- *Open ended answer*

**Participant Pre-Visit Questionnaire**

1*.* What time did you wake up this morning (24:00)? (Round up to nearest hour)

- *Open ended answer*

2. Did you do any near work within 1 hour prior to this appointment?

- *No*
- *Yes*

3. Did you have any caffeine-containing drinks (e.g., coffee or energy drinks) prior to this appointment?

- *No*
- *Yes (Answer 3a & 3b)*

3a. How much caffeine did you drink (estimate in cups or bottles)?

- *Open ended answer*

3b. What time (24:00) did you drink caffeine-containing drinks? (Round to nearest hour)

- *Open ended answer*

4. Did you drink any alcohol/kombucha prior to this appointment?

- *No*
- *Yes (Answer 4a & 4b)*

4a. How much alcohol/kombucha did you drink (estimate in cups or bottles)?

- *Open ended answer*

4b. What time (24:00) did you drink alcohol/kombucha this morning (round to nearest hour)?

- *Open ended answer*

5. Did you exercise this morning?

- *No*
- *Yes (Answer 5a)*

5a. For how long did you exercise and what time (24:00)?

- *Open ended answer*

6. Did you use any eye drops this morning?

- *No*
- *Yes (Answer 6a & 6b)*

6a. If you used eye drops this morning, what kind?

- *Open ended answer*

6b. What time did you use the eye drops this morning (24:00)?

- *Open ended answer*

*7.* Did you take any systemic medications this morning?

- *No*
- *Yes (Answer 7a & 7b)*

7a. What kind of systemic medications did you take this morning?

- *Open ended answer*

7b. What time did you take your systemic medications this morning (24:00)?

- *Open ended answer*

8. What time did your put your contact lenses this morning (24:00)?

- *Open ended answer*
